# Evaluating the Spatiotemporal Characteristics of Agricultural Eco-Efficiency Alongside China’s Carbon Neutrality Targets

**DOI:** 10.3390/ijerph192315478

**Published:** 2022-11-22

**Authors:** Changming Cheng, Jieqiong Li, Yuqing Qiu, Chunfeng Gao, Qiang Gao

**Affiliations:** 1 College of Economics and Management, Nanjing Forestry University, Nanjing 210037, China; 2 School of Economics and Management, Chuzhou University, Chuzhou 239000, China; 3 Science and Technology Department, Nanjing Forestry University, Nanjing 210037, China; 4 School of Urban Economics and Management, Beijing University of Civil Engineering and Architecture, Beijing 100044, China

**Keywords:** agricultural eco-efficiency, agricultural carbon emissions, agricultural carbon sequestration, slack-based modeling, undesirable outputs, carbon neutrality target

## Abstract

Agriculture has the dual effect of contributing to both carbon emissions and sequestration, and thus plays a critical role in mitigating global climate change and achieving carbon neutrality. Agricultural eco-efficiency (AEE) is an important measurement through which we can assess the efforts toward reduced emissions and increased sequestration. The purpose of this study was to understand the relationship between China’s target of carbon neutrality and AEE through an evaluative model, so as to improve AEE and ultimately achieve sustainable agricultural development. The Super-SBM model scientifically measures the AEE based on provincial panel data collected between 2000 and 2020. We selected kernel density function and spatial distribution to explore the spatial and temporal evolutionary trends, and used a Tobit model to identify the drivers of AEE. The research shows that (1) China’s agricultural system functions as a net carbon sink, with all provinces’ agricultural carbon sequestration levels recorded as higher than their carbon emissions from 2000 to 2020. (2) Despite sequestration levels, the level of AEE in China is not high enough, and the average efficiency level from 2000 to 2020 is 0.7726, showing an overall trend where AEE decreased at first and then increased. (3) The AEE of each province is clearly polarized; there are obvious core–periphery characteristics and spatial distribution of clustered contiguous areas. Central provinces generally have lower efficiency, eastern and northeastern provinces have higher efficiency, and northeastern provinces always remain in the high-efficiency group. (4) Influencing factors show that urbanization, upgrading of industrial structure, financial support for agriculture, and mechanization have a significant positive impact on AEE. These findings have important implications for the promotion of the low-carbon green development of Chinese agriculture.

## 1. Introduction

Climate change is a global concern as it causes extreme weather and destroys natural resources such as glaciers, which negatively impacts economic and social development. Increasing CO_2_ emissions are considered to be an important factor contributing to global warming [1,2,3]. According to the data from ITA, China will emit more than 11.9 billion tons of CO_2_ in 2021, accounting for 33% of total global emissions [4]. As an active participant and supporter of global climate governance, China actively implements green development strategies and is committed to energy conservation, emission reduction, and environmental protection [5,6,7]. With these goals in mind, the Chinese government announced that it would strive to reach peak carbon emissions by 2030 and achieve carbon neutrality by 2060, which is a significant contribution toward achieving the global carbon neutrality target [2]. China’s agricultural carbon emissions account for 17% of the country’s carbon emissions; comparatively, with the global average measured at 11% [8]. This makes China the world’s largest emitter of carbon from agriculture, and further demonstrates why the goal of carbon neutrality cannot be achieved without the full engagement of the agricultural sector [9].

The agricultural sector not only emits greenhouse gases, but also functions as a carbon sequestration sink [10,11,12]. Greenhouse gas emissions in agricultural production mostly emanate from practices such as tillage, irrigation, use of agricultural materials, and the cultivation of rice [13,14], and 13% of global CO_2_, 44% of CH_2_, and 82% of N_2_O emissions come from agricultural systems [3]. On the other hand, specific agricultural practices and spaces can absorb large amounts of CO_2_ through photosynthesis, such as forestry, crop growth, and pastured land, which all have a significant carbon sequestration capacity [15,16]. China has a large population and the area of arable land per capita is small. The quality of this land is not high, and agricultural growth has long relied on the excessive use of chemicals [17,18,19], which has severely overstretched the available arable land and produced large amounts of carbon emissions. These issues demonstrate how serious the challenges are for the agricultural eco-system [20,21]. However, the proper use of cropland can effectively reduce carbon emissions and increase carbon sequestration [22], and studies have shown that Chinese agriculture is a huge carbon sequestration system with significant potential to promote carbon neutrality [23].

The Chinese government places great importance on promoting the agricultural reduction in emissions and increasing sequestration. The Opinions on the Complete and Accurate Implementation of the New Development Concept and the Good Work of Carbon Peaks and Carbon Neutrality in October 2021 proposed accelerating the development of green agriculture, promoting the sequestration of carbon, and enhancing ecological agricultural carbon sequestration. Likewise, China’s No.1 Central Document 2022 proposed to research and apply agricultural technologies to reduce carbon, increase carbon sinks, and explore research into the value of carbon sink products. Therefore, in the context of carbon neutrality, exploring the balance between economic growth and low-carbon agricultural development through green and sustainable development is an effective means in China. An effective action towards sustainable development requires a scientific measurement of current AEE and an analysis of spatial and temporal differences and driving factors, in order to explore appropriate paths for improving AEE.

AEE under the carbon neutral target is an effective tool to reflect the effectiveness of green and low-carbon agricultural development, and this refers to the practice of generating as much agricultural output with as little environmental pollution as possible in order to achieve concurrent economic and environmental benefits [24,25,26]. At present, many scholars have conducted research on how to measure AEE, and at the core of this research is the selection of indicators and the construction of models [27,28]. When selecting indicators, undesirable outputs should be included in the research framework. Scholars usually consider agricultural pollution emissions to be undesirable outputs, including pollutants such as greenhouse gas emissions, chemical oxygen demand, and so on [29,30,31], and agricultural carbon emissions are adopted by most scholars [32]. Desired outputs are mainly measured using agricultural added value or crop yields. Model construction mainly includes the SFA model, the DEA model, and their derivative models, such as the SBM model, the Super-SBM model, and the directional distance function (DDF) model [33,34,35,36]. Since SFA models can only use one output and have difficulty taking undesirable outputs into account [37], non-radial, non-angular SBM models are widely used in agroecological efficiency measurements, because they have the ability to handle undesirable outputs [38,39]. Existing research further explores the influencing factors of AEE [38,40].

Currently, existing studies have achieved useful results, but there are some limitations. Firstly, most studies have taken into account the undesired output factor of carbon emissions in agricultural production, but agriculture also has the attribute of carbon sequestration, and there is a lack of systematic studies that integrate agricultural carbon emissions and sequestration into the same analytical framework. Secondly, most existing studies are confined to the measurement of AEE and lack in-depth analysis of the contributing spatial and temporal characteristics and influencing factors of eco-efficiency, making it difficult to accurately explore effective countermeasures to improve AEE. To address these issues, the Super-SBM model is used in this research, which includes carbon emissions and sequestration, to conduct an empirical study of AEE throughout China. On the basis of scientific measurements of AEE, we further examine the spatial and temporal characteristics and influencing factors of AEE in China, and attempt to expand the current literature in the following ways: (1) Effectively measure agricultural carbon emissions (material inputs, rice fields, and soils) and carbon sequestration (crops) per province from 2000 to 2020 and explore the dynamic evolutionary characteristics. (2) Construct a Super-SBM model including carbon emissions and carbon sequestration to measure AEE in China, recording AEE by province and by region (eastern, northeastern, central, and western China), and exploring spatial and temporal evolutionary trends using kernel density functions and spatial distributions. (3) Analyze the influencing factors of AEE in China with the help of a panel Tobit regression model, and screen effective paths to improve AEE.

The rest of the study is arranged as follows. Section 2 covers the data and methods used in this paper. Section 3 analyzes agricultural carbon emissions and sequestration, the spatiotemporal characteristics of AEE, and the influencing factors of AEE. Section 4 discusses the conclusions and policy implications.

## 2. Materials and Methods

### 2.1. Accounting for Agricultural Carbon Emissions and Sequestration

Based on current research [3,41,42], this paper examines agricultural carbon emissions from three perspectives, namely agricultural materials, rice fields, and soil (Table 1). Specifically, (1) the use of agricultural chemicals, including fertilizers, pesticides, agricultural films, agricultural diesel fuel, and irrigation activities that consume electricity, will produce carbon emissions (Appendix A for coefficients); (2) rice field CH_4_ emissions include data from rice growth periods because, due to different hydrothermal conditions in different parts of China, CH_4_ emissions during the growth cycle may vary. Taking into account the soil, climate, and hydrological conditions of rice cultivation, the emission coefficients of CH_4_ for early-season rice, mid-season rice, and late-season rice in different provinces were determined and used to calculate the CH_4_ emissions from rice fields (Appendix B for details of the coefficients); (3) during crop cultivation, soil layer destruction causes N_2_O emissions. We took measurements of N_2_O emissions specifically from rice, spring wheat, winter wheat, soybeans, corn, and vegetables (Appendix C for details of the coefficients).

Agricultural carbon sequestration mainly measures the carbon uptake by crops, that is, the carbon dioxide absorbed by crops through photosynthesis during their lifecycle, which is one of the important sources of carbon sequestration [45]. Based on relevant studies, the total agricultural carbon sequestration in this study is calculated using the following formula:

(1) $$Cs=\sum_{j=1}^{n}Cs_{i}=\sum_{j=1}^{n}\left\{\left[C_{j}\times Y_{j}\times \left(1-W_{j}\right)\right]/H_{j}\right\}$$

where Cs is the total carbon sequestration and $Cs_{j}$ is the carbon sequestration of j. For the same crop, $C_{j}$ is the carbon content rate, $Y_{j}$ is the economic yield, $W_{j}$ is the moisture factor, and $H_{j}$ is the economic factor. Details of the parameters are shown in Appendix D. According to prior research of [46], the effect of 1 t CH_4_ and N_2_O is equivalent to 25 t CO_2_ (6.8182 t C) and 298 t CO_2_ (81.2727 t C), which are all converted to C emissions later.

### 2.2. Methodology Specification

#### 2.2.1. Measuring AEE: Super-SBM Model

During agricultural production, alongside any expected economic benefits, there are factor inputs that accompany undesirable outputs, but it is vital to limit these outputs [38]. As traditional DEA models suffer from input factor “crowding” or “slack”, they tend to produce biased results and are unable to deal with undesirable output indicators [47]. The SBM model is capable of considering unexpected outputs and can effectively compensate for the shortcomings of traditional DEA models. However, the SBM model has an issue consistent with the traditional DEA model, in that it is also unable to distinguish between decision units (DMUs) that are both efficient at 1. On this basis, the Super-SBM model is used in this paper to measure AEE, which is thus able to deal with undesirable output, and further compare and differentiate efficient DMUs that are on the frontier [48]. The model is constructed as follows:

(2) $$\rho =\min\frac{\frac{1}{m}\sum_{i=1}^{m}\frac{\bar{x}}{x_{ik}}}{\frac{1}{s_{1}+s_{2}}(\sum_{r=1}^{s_{1}}\frac{{\bar{y}}^{g}}{y_{rk}^{g}}+\sum_{t=1}^{s_{2}}\frac{{\bar{y}}^{b}}{y_{tk}^{b}})}$$

(3) $$s.t.\left\{\begin{array}{l} \bar{x}\geq \sum_{j=1,j\neq k}^{n}\lambda_{j}x_{ij},{\bar{y}}^{g}\leq \sum_{j=1,j\neq k}^{n}\lambda_{j}y_{rj}^{g},{\bar{y}}^{b}\geq \sum_{j=1,j\neq k}^{n}\lambda_{j}y_{tj}^{b}\\ \bar{x}\geq x_{k},{\bar{y}}^{g}\leq y_{k}^{g},{\bar{y}}^{b}\geq y_{k}^{b},\lambda_{j}\geq 0\\ i=1,2,\cdots ,m;j=1,2,\cdots ,n\\ r=1,2,\cdots ,s_{1},t=1,2,\cdots ,s_{2} \end{array}\right.$$

where ρ is the value of AEE, n is the number of DMUs, m is the number of inputs, $r_{1}$ is the number of desirable outputs, and $r_{2}$ is the number of undesirable outputs. Vectors x, $y^{g}$, and $y^{b}$ represent inputs, desirable outputs, and undesirable outputs. When ρ≥1, the AEE of the target decision unit is relatively effective; when ρ<1, the AEE of the target decision unit has not reached efficiency, and there is redundancy or a shortage of inputs or outputs.

#### 2.2.2. Inspect the Dynamic Evolution Characteristics: Kernel Density Estimation

As a kind of non-parametric probability density estimation, kernel density estimation is a common method for the study of disequilibrium distribution, which can describe the distribution pattern of random variables through continuous density curves using kernels as weights [49]. The changes in the distribution pattern of the kernel density curve, changes in kurtosis, and changes in the location of the curve can be analyzed to reveal the dynamic evolution characteristics of AEE. In this paper, we choose the Gaussian kernel, which is commonly used in existing studies, and the calculation formula is as follows:

(4) $${\hat{f}}_{h}(x)=\frac{1}{n}\sum_{i=1}^{n}K_{h}\left(x-x_{i}\right)=\frac{1}{nh}\sum_{i=1}^{n}K\left(\frac{x-x_{i}}{h}\right)$$

where $x_{1},x_{2},\cdots ,x_{i}$ is an independent distribution of n sample points, $K\left(x\right)$ is a random kernel function, and the magnitude of the bandwidth h value affects the smoothness of the kernel density curve distribution.

#### 2.2.3. Verifying the Influencing Factors: Tobit Model

The AEE measured in this paper using the Super-SBM model is non-negative truncated data, representing a restricted dependent variable. Therefore, using OLS estimation would lead to biased results [50]. To solve this issue, the Tobit model proposed by the American economist Tobin in 1958 is a suitable choice [51], so we used the Tobit model to construct an econometric model of the factors influencing AEE, which is calculated as follows:

(5) $$y_{it}=\left\{\begin{array}{ll} \beta^{T}x_{it}+\varepsilon_{it} & \beta^{T}x_{it}+\varepsilon_{it}>0\\ 0 & otherwise \end{array}\right.$$

where $y_{it}$ is the AEE of the i province in the t year; $x_{it}$ is the explanatory variable, which refers to the factors influencing AEE; $\beta^{T}$ is the regression coefficient of the explanatory variable; and $\varepsilon_{it}$ is a random error term subject to $N\left(0,\sigma^{2}\right)$.

### 2.3. Data

#### 2.3.1. Data Description

**Figure 1:**
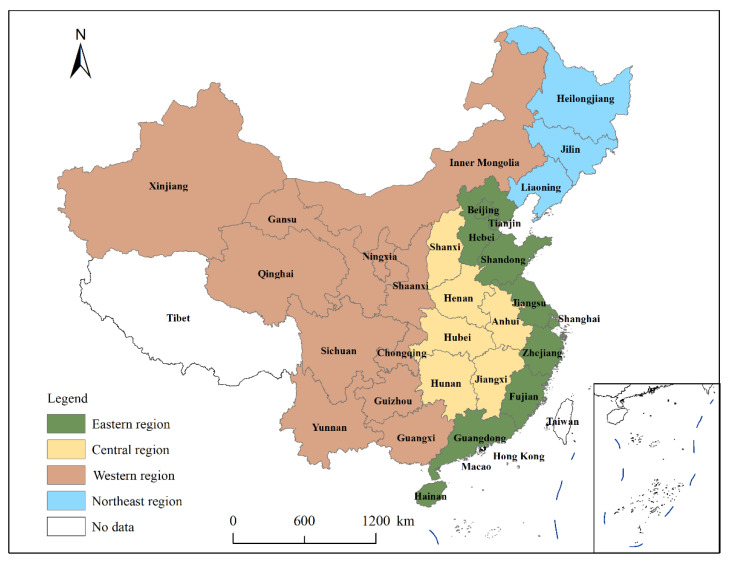
Study areas.

The study area of this paper covers 30 provinces in China, excluding Hong Kong, Macao, Taiwan, and Tibet, given the availability of data. The study provinces are divided into four regions according to the Chinese statistical partitioning criteria (Figure 1). This paper uses the data of 30 provinces from 2000 to 2020, and the required data include an account of agricultural carbon emissions and sequestration, inputs and outputs of AEE, and its influencing factors. Data were obtained from the China Statistical Yearbook, China Agricultural Yearbook, China Rural Statistical Yearbook, China Population and Employment Statistical Yearbook, China Agricultural Products Import and Export Monthly Statistical Report, and provincial and municipal statistical yearbooks, and any missing data were filled in by the interpolation method.

#### 2.3.2. Evaluation Indicators

Agriculture covers a wide range of practices, including crop farming, forestry, animal husbandry, fish farming, and sideline industries. Because each subsector varies greatly between geographic regions, agriculture in this paper is limited to the category of the plantation industry in an effort to elevate the relevance and specificity of the study. The measure of AEE under the carbon neutrality target includes inputs, desirable outputs, and undesirable output. Five input indicators, two desirable output indicators, and one undesirable output indicator are constructed by referring to the studies of related scholars [3,30,52,53]. The input indicators include labor, land, machinery, fertilizer, and irrigation, where agricultural employment = regional primary industry employment x (value added in agriculture/value added in the primary industry). Desirable outputs are expressed in terms of gross agricultural output and agricultural carbon sequestration, and the output value is converted into comparable data. The undesirable output choices agricultural carbon emissions, which are measured by agricultural materials, rice fields, and soil carbon emissions. The indicators and descriptive statistics are shown in Table 2.

#### 2.3.3. Influencing Factors on AEE

Another focus of this study was to explore the influencing factors of AEE under the carbon neutrality target. The goal of the study is to provide information that will promote low-carbon sustainable development in agriculture, and to enable agriculture to play a more prominent role in promoting the carbon neutrality target. Based on the usefulness and validity of the data, we constructed the influencing factors driving AEE from the perspectives of economic foundations, production conditions, agricultural support policies, and technological innovation [53,54,55,56,57]. (1) Economic conditions were measured using the urbanization rate (URBAN) and the industrial structure upgrading index (ISU). (2) Production conditions were measured using the agricultural cultivation structure (ACS), the degree of agricultural disaster (DISA), and the multiple crop index (MCI). (3) Agricultural support policies were measured via the annual financial support for agriculture (FSFA). (4) The mechanization level (MECH) measured from the perspective of technological innovation. Details of each measurement are shown in Table 3.

## 3. Results and Analysis

### 3.1. Analysis of Agricultural Carbon Emissions and Sequestration in China

**Figure 2:**
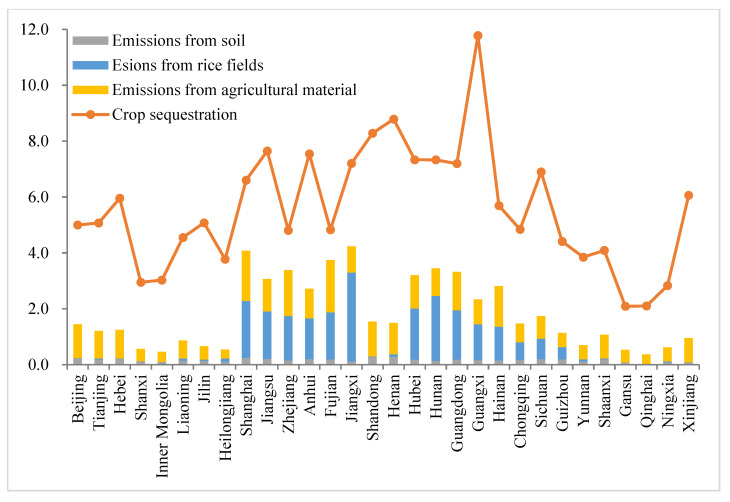
Agricultural carbon emissions and sequestration in China from 2000 to 2020 (t/hm^2^).

Using the above calculation list of agricultural carbon emissions and sequestration, the emissions and sequestration per hectare of farmland were estimated for 30 provinces in China; the results are shown in Figure 2.

The agricultural carbon sequestration across all provinces in China from 2000 to 2020 is higher than the carbon emissions, indicating that agricultural systems across all provinces belong to the net carbon sink [58], with an average net carbon sequestration of 3.754 t/hm^2^. This finding is consistent with the results of existing studies [3]. Specifically, the national average agricultural carbon sequestration is 5.585 t/hm^2^, among which 11 provinces, including Guangxi (11.778 t/hm^2^), Henan (8.878 t/hm^2^), Shandong (8.820 t/hm^2^), Jiangsu (7.645 t/hm^2^), and Anhui (7.543 t/hm^2^), have an average agricultural carbon sequestration higher than the national level. These provinces are all large agricultural provinces, with crop cultivation areas ranking among the top in China, such as sugar cane in Guangxi, wheat in Henan, vegetables in Shandong, and rice in Jiangsu. The agricultural carbon sequestration of Gansu, Qinghai, Ningxia, and Shaanxi are at the bottom of the list, all below 0.3 t/hm^2^.

As a comparison to carbon sequestration, the national average carbon emission intensity is 1.831 t/hm^2^, of which 0.923 t/hm^2^ is attributed to agricultural materials, 0.750 t/hm^2^ emanates from paddy fields, and 0.158 t/hm^2^ emanates from soils. This demonstrates that agricultural materials are the main contributor to carbon emissions, especially fertilizer, diesel, and agricultural films. The intensity of agricultural carbon emissions in most provinces is distributed between 1 and 4 t/hm^2^. Eight provinces, including Jiangxi (4.233 t/hm^2^), Shanghai (4.074 t/hm^2^), and Fujian (3.740 t/hm^2^), contribute the greatest amount to carbon emissions, with measurements all greater than 3 t/hm^2^. Contrastingly, 10 provinces, including Qinghai (0.365 t/hm^2^), Inner Mongolia (0.457 t/hm^2^), and Gansu (0.527 t/hm^2^), ranked lowest with less than 1 t/hm^2^ of recorded carbon emissions. The sources of carbon emissions vary greatly by province, with carbon emissions from rice paddies exceeding 60% in Jiangxi and Hunan, and agricultural carbon emissions accounting for over 90% in Qinghai and Xinjiang, while soil carbon emissions are low in all provinces, with the highest, Shanxi, accounting for only 21.7%. The provinces with high agricultural carbon emissions are mainly located in the central region where there are differences in agricultural operations and resource use, and the transition to low-carbon agriculture may require a differentiated approach.

### 3.2. Evaluation and Analysis of AEE in China

**Figure 3:**
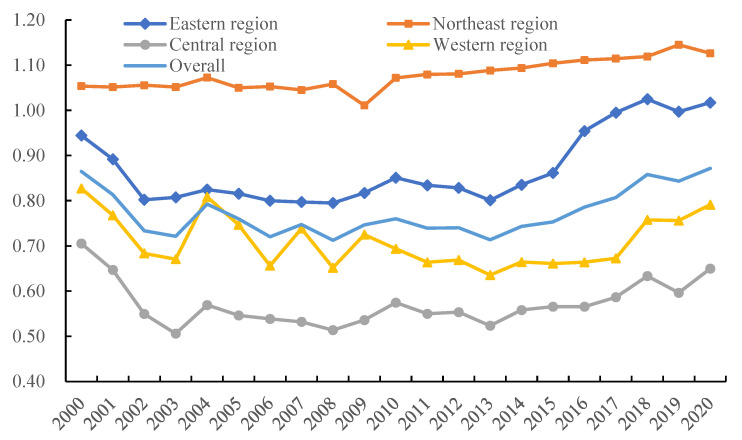
Evolution of AEE in China and four regions from 2000 to 2020.

The Super-SBM model for undesirable outputs was applied, based on MATLAB2020b software, to evaluate the AEE per province from 2000 to 2020 under the carbon neutrality target. The results are shown in Table 4 and Figure 3.

#### 3.2.1. Overall evolution of the AEE

In Figure 3, we can observe that China’s AEE declined but was followed by an increase between 2000 and 2020. The national average value of AEE in 2000 was 0.8645, followed by an oscillating decline, reaching the lowest value of 0.7125 in 2008, and then slowly rising to 0.8718 in 2020, with an overall upward U-shaped distribution. In terms of regional differences, the AEE value of the northeast region (1.0778) maintained its lead, ranking far above the national average, which has already reached an effective state, and showing a slow upward trend from 2000 to 2020 with an increase of 6.9% overall. In the eastern region, the AEE value presents a U-shaped change, with slowly declining rates from 2000 to 2008, which then fluctuate upward, reaching an efficiency value of 1.0171 in 2020. In the western region, AEE values fluctuated greatly from 2000 to 2009, and then much like the eastern region, shifted toward a trend of first decreasing and then increasing, with an average efficiency value of 0.7096 from 2000 to 2020. The central region has the lowest mean value of AEE at 0.5713. Overall, there are clear regional differences in AEE values; the efficiency of the northeast region is the highest, followed by the eastern and western, and the central region is the lowest.

#### 3.2.2. Inter-Provincial Variation in AEE

Ten provinces, accounting for 33.3% of all provinces, have mean AEE values above the national average from 2000 to 2020: Jilin, Heilongjiang, and Liaoning in the northeast; Hainan, Beijing, Shanghai, Guangdong, and Jiangsu in the east; and Guangxi and Xinjiang in the west. Among them, eight provinces, including Hainan and Beijing, have efficiency averages above 1 and have reached the DEA effective status in the vast majority of years. Ningxia, Shanxi, Anhui, Hunan, Hubei, Gansu, Shaanxi, and Jiangxi have relatively low AEE, with mean values below 0.6, and all belong to the central and western regions, with Ningxia having the lowest efficiency value, at a mean value of 0.4628, which is only 39.2% of the mean value of the highest-ranking province, Hainan, and 59.9% of the mean value of the national average. It can be seen that from the perspective of geographic zones, the AEE of some provinces in the central and western regions is not high enough. In the future, emphasis in these regions should be placed on the integrated development of resource allocation and low-carbon transformation, the establishment of a synergistic development mechanism through exerting the demonstration effect and catch-up effect, and the improvement of the overall level of AEE.

### 3.3. Spatiotemporal Characteristics of AEE in China

#### 3.3.1. Kernel Density Estimation of AEE

**Figure 4:**
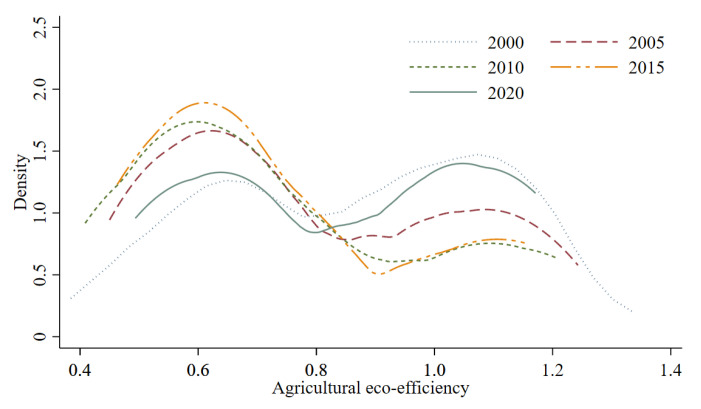
Kernel density of AEE in major years.

Using Stata16 software, the Kernel density function was applied to estimate the AEE values under the carbon neutrality target in 2000, 2005, 2010, 2015, and 2020, and a kernel density curve was drawn (Figure 4). The investigation of the dynamic evolution characteristics yielded the following observations. (1) From the position of the center of gravity of the kernel density curve, which shifts left and then right from 2000 to 2020, it is clear that China’s inter-provincial AEE under the carbon neutrality target first declines and then increases. (2) From the shape of the kernel density curves, where all five curves show the coexistence of the main peak and the secondary peak, we see the suggestion of a significant pattern of polarization in AEE. The height of the main peak gradually increases from 2000 to 2015, and the gap between provinces gradually widens, while the gap between the peaks decreases and flattens out in 2020; this indicates that the AEE gap among provinces narrowed and the degree of polarization was reduced. (3) From the trailing edge of the kernel density curve, it can be noted that the left and right sides of the corresponding curves in 2000 and 2020 are similar, while the trailing edge on the right side of the corresponding curves in 2005, 2010, and 2015 is longer than the trailing edge on the left side. This shift shows a clustering of low values of AEE under the carbon neutrality target in this period. This demonstrates that the AEE of different provinces in China has different patterns in different periods, with different dynamic evolutionary characteristics such as development level and polarization degree.

#### 3.3.2. Spatial Distribution Patterns of AEE

**Figure 5:**
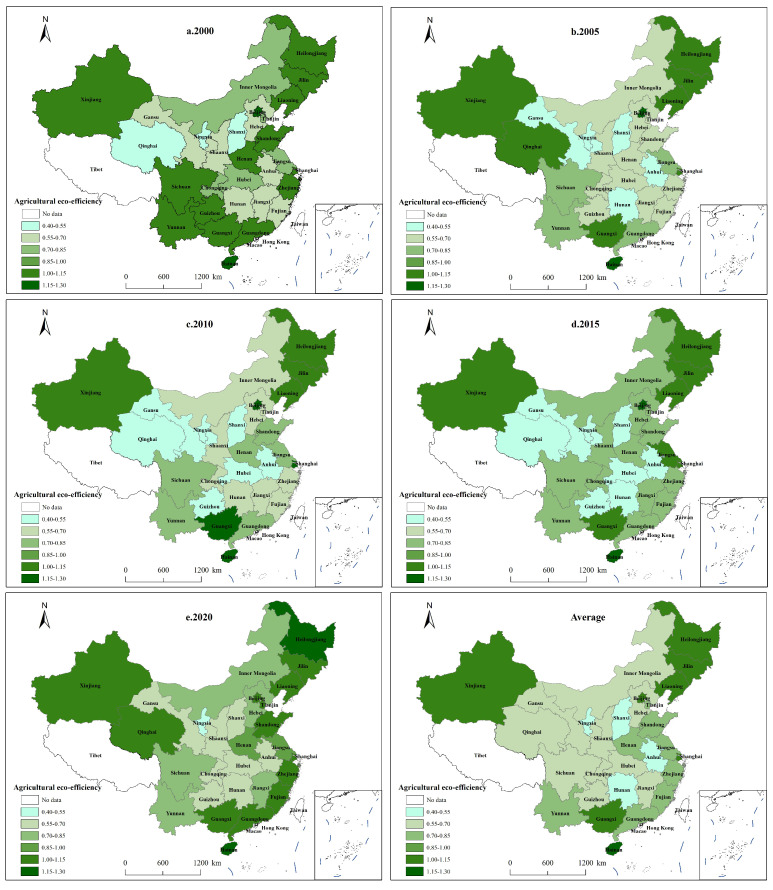
Spatial distribution pattern of AEE in China.

To visualize the spatial characteristics of AEE per province, this paper uses ArcGIS 10.8 software to create a visual map of AEE at five time points and an average point, which is shown in Figure 5.

Figure 5 demonstrates that during the study period, China’s AEE first decreased, then increased, and had obvious spatial distribution characteristics of clustering and contiguity. In 2000, AEE was relatively high, with 15 provinces having efficiency values greater than 1, accounting for 50%. These provinces were mainly concentrated in the northeast, but also included most of the eastern and some parts of the western regions, whereas low efficiency was concentrated in the central region, which again shows high efficiency in the north and south and low efficiency in the central region in terms of spatial distribution. The years 2005, 2010, and 2015 saw a decline in AEE, with the number of efficient provinces decreasing. The number of provinces with AEE over 1 fluctuated to nine, eight, and nine again, respectively, while the number of provinces with AEE less than 0.7 likewise fluctuated but showed an increase, shifting between 17, 16, and 18, respectively, with a particularly marked decline in the central and western regions, showing a spatial distribution characterized by the clustering of low values. In 2020, AEE improved considerably, with only one province, Ningxia, having an efficiency value below 0.55, and with the number of provinces with AEE above 1 rising to 14. These regions are mainly clustered in the northeast and eastern regions, with a spatial distribution characterized by a concentration of high values and a gradual narrowing of the gap between regions.

As a whole, the spatial distribution of AEE in China has obvious core–periphery characteristics, whether from the perspective of five time points or the average efficiency distribution map from 2000 to 2020. As established, the efficiency of the central provinces is generally lower, while the eastern and northeastern provinces have higher efficiency rates, with the northeastern provinces always in the high-efficiency group. With its flat terrain, fertile resources, good lighting, and relatively high level of scale and intensification, the northeastern region is more conducive to promoting a low-carbon transition in agriculture and achieving a balance between carbon neutrality and agricultural production. The eastern region is economically developed to achieve more efficient and low-carbon operations while ensuring agricultural output via advanced technological tools and a strong policy environment. The western and central regions, however, have a relatively poor economic foundation, technological support has yet to be strengthened, and agricultural production needs to be transformed from extensive to refined.

### 3.4. Analysis on the Influencing Factors of AEE in China

When using the Tobit model, we can determine whether fixed or random effects should be used by testing for the presence of individual effects. The individual and random errors in the model are small, the variance ratios ρ are above 0.5, and the individual effect variances are large and all pass the LR test, strongly rejecting the original hypothesis. The Wald test passes the 1% significance test and the model worked well, so it is reasonable to use the random effects panel Tobit model.

According to Table 5, the coefficients of all variables pass the 1% or 5% significance level test. URBAN, ISU, FSFA, and MECH have positive effects, while ACS, DISA, and MCI have negative effects.

This summary more specifically means the following:

(1) In relation to a region’s economic foundation, the urbanization rate positively affects AEE at the 1% significance level. This is mainly due to the fact that although urbanization brings about the loss of arable land and labor migration, it also induces a transition to more efficient specialization in agriculture as it increases the scarcity of inputs. Furthermore, agricultural productivity increases due to a rise in technological progress and the transformation of the industrial structure, which is brought about by urbanization [59]. The coefficient of ISU is 0.154, which significantly and positively affects AEE. This is because the industrial structure is optimized and upgraded, so the cluster effect and specialization effect gradually emerge, which not only reduces agricultural production costs and increases the added value of products, but also brings huge structural and scale dividends, which further help improve AEE [53].
(2) In terms of production conditions, cropping structure negatively affects AEE at a significance level of 5%, which is consistent with the findings of [38], and rejects the assertion that cash crops increase the burden on the environment, and that it is feasible to moderately adjust agricultural cropping structure on the basis of ensuring food security. Natural disasters not only affect agricultural acreage and reduce crop yields and agricultural output, but also drive ecological degradation, which has a significant negative impact on AEE gains. The replanting coefficient, which reflects the intensity of cultivation of arable land, can increase agricultural value added, but can also bring about an increase in the number of tillage and chemical inputs. The replanting coefficient also requires an increase in input intensity, thereby bringing about an increase in undesired agricultural output, which likewise demonstrates the unsustainability of long-term and high-intensity cultivation [60].
(3) As far as agricultural support policies are concerned, the financial support from the government for agriculture comes mainly in the form of investment and subsidies, which can support the construction of agricultural infrastructure and improve the input structure of agricultural production, thus enhancing AEE.
(4) Regarding technological innovation, the widespread use of agricultural machinery can enhance production technology [61], resulting in improved productivity and production efficiency. However, increased machinery brings an increase in the use of petrochemical resources, so it is necessary to preferentially use low-carbon, green, and efficient agricultural machinery first.

## 4. Discussion and Policy Implications

Based on the measurement of agricultural carbon emissions and sequestration, this paper has explored the spatiotemporal characteristics of AEE as well as its influencing factors. The results from this study suggest, compared with rice fields and soil, that agricultural materials generate relatively large carbon emissions, which is consistent with [3]. Therefore, agricultural emission reduction and efficiency enhancement need to pay special attention to the control of agricultural material input. China’s AEE has obvious core–periphery characteristics and shows obvious regional differences. This means that a one-size-fits-all policy is no longer applicable, and it is necessary to balance the distribution of resources in different regions while improving the expected output. The main objectives are to reduce the external inputs, increase agricultural output value and carbon sink level, and reduce agricultural carbon emissions.

On the basis of the findings above, we make the following policy recommendations:

(1) Deploy differentiated initiatives to reduce emissions and increase sequestration in agriculture. Specifically, work to improve the utilization rate of agricultural inputs [62], particularly in provinces such as Qinghai and Xinjiang, where the share of carbon emissions from agricultural inputs is relatively high. This can be achieved by encouraging the application of organic fertilizers and soil testing fertilizers, while promoting the resourceful use of straw. While the agricultural system works largely as a carbon sink, the agricultural cultivation structure should be further optimized to strengthen the carbon sequestration role of crops.
(2) Assess the AEE under the carbon neutrality targets in each region, and change the behavior cease of pursuing high efficiency while ignoring environmental constraints. Agricultural carbon emissions and carbon sequestration must be central to the future research framework and focus on the balance between economy and environment. Furthermore, policies to promote AEE should be formulated in accordance with local conditions with consideration of regional differences in resource endowments, industrial structures, and economic bases. Agricultural carbon emission constraints should be made a government planning target, and any agricultural subsidy policies oriented towards green and low-carbon development should be constructed to cultivate and promote green agricultural technologies, so as to achieve a win-win situation for both environmental protection and effective allocation of scientific and technological resources.
(3) The current problems of low AEE and regional imbalances require the development of cooperation plans for inter-regional collaboration, which must be formulated to balance the distribution of poorer and wealthier regional resources, to strengthen the supervision and management of resource elements, and to improve the allocation performance of various types of resources for AEE. The central and western regions have the opportunity to make large strides in the promotion of AEE by increasing investment in scientific research and by strengthening collaborative innovation [63]. The eastern and northeastern regions should continue to improve the level of resource allocation, increase research and development around core technologies, and play a leading role in the achievement of balanced and integrated AEE practices through active exchange and cooperation.

There are some shortcomings to this research. Firstly, our study only estimates agricultural carbon emissions and carbon sequestration from the narrow perspective of plantation agriculture, and lacks measurements that cover the broader scope of agricultural practices. Secondly, the coefficients for agricultural carbon accounting were obtained from the list published by the Chinese government and academic literature, but more accurate measurements could be taken, so there are some uncertainties around the current coefficients, thus affecting the reliability of the results to a certain extent. Thirdly, due to data availability constraints, this study lacks small-scale studies at the municipal or county level, and in the future, consideration will be given to a wider range of smaller-scale research units to improve accuracy.

## 5. Conclusions

The strategic goal of carbon neutrality has placed higher demands on the green and low-carbon development of agriculture in China. In this paper, we incorporated agricultural carbon emissions and sequestration into the model to build an AEE measurement model under the carbon neutrality target. On this basis, the Super-SBM model was employed to measure the AEE per province in order to assess the efforts toward reduced emissions and increased sequestration. Furthermore, we analyzed the spatial and temporal characteristics of AEE, and used the Tobit model to investigate the factors influencing AEE. The results of this study are as follows:

(1) China’s agricultural system functions as a net carbon sink, with the agricultural carbon sequestration of all provinces from 2000 to 2020 measuring at higher rates than the carbon emissions. The national average carbon sequestration is 5.585 t/hm^2^ and the average net carbon sequestration is 3.754 t/hm^2^. Considering the national average carbon emission intensity of 1.831 t/hm^2^, including 0.923 t/hm^2^ for agricultural materials, 0.750 t/hm^2^ for paddy fields, and 0.158 t/hm^2^ for soils, it is clear that the use of agricultural materials is the main source of carbon emissions from agriculture.
(2) From 2000 to 2020, the national average AEE was not high enough, with an average value of 0.7726, showing a trend of decreasing and then increasing, and there is still much room for improvement. In terms of spatial distribution, China’s AEE has obvious core–periphery characteristics and shows a clustered and contiguous spatial distribution, with central provinces generally having lower efficiency, eastern and northeastern provinces having higher efficiency, and northeastern provinces always in the high-efficiency group.
(3) As for the influencing factors, urbanization, upgrading of industrial structure, financial support for agriculture, and mechanization can significantly contribute to AEE, with urbanization and financial support for agriculture having a greater degree of influence. In contrast, agricultural cultivation structure, agricultural disaster, and replanting have a negative impact on the AEE.

## Figures and Tables

**Table 1:** Agricultural carbon emissions and sequestration.

Carbon Effect	Category	Cause	Factor	Data Required	Reference
Carbon Emissions	Agricultural materials	The production, application, and decomposition of fertilizers lead to carbon emissions.	Fertilizer	Consumption of fertilizer	[41]
		The production, application, and decomposition of pesticides lead to carbon emissions.	Pesticide	Consumption of pesticide	
		The production, application, and decomposition of mulches lead to carbon emissions.	Agricultural film	The amount of agricultural film used	
		The consumption of diesel by machinery leads to carbon emissions.	Diesel	Diesel oil used in agriculture	
		The fossil fuels consumed for generating electricity in irrigation result in carbon emissions indirectly.	Irrigation	Effective irrigation area	
	Rice fields	Methanogens in rice fields utilize organic matter from the roots of rice plants to form methane.	Rice field	Planting area of early rice, medium rice, late rice	[43,44]
	Soil	Soil surface releases carbon when planting crops.	Soil	Yields of rice, winter wheat, spring wheat, soybeans, maize, vegetables	[42]
Carbon Sequestration	Crop sequestration	Crops absorb carbon dioxide through photosynthesis.	Crop	Yield of various crops, such as rice, wheat, maize, pulses, vegetables	[42]

**Table 2:** Input and output variables for the measure of AEE.

Type	Variable	Explanation	Units
Input indicators	Labor	The number of agricultural practitioners	10^4^ person
	Land	Total sown areas of crops	10^3^ ha
	Machinery	Total power of agricultural machinery	10^4^ kW
	Fertilizer	Application quantity of chemical fertilizer	10^4^ t
	Irrigation	Effective irrigation area	10^3^ ha
Output indicators	Desirable output	Actual output value of agriculture	10^8^ CNY
		Agricultural carbon sequestration	10^4^ t
	Undesirable output	Agricultural carbon emissions	10^4^ t

**Table 3:** Influencing factors of AEE growth.

Variables	Description	Mean	Std. Dev.	Min	Max
URBAN	Urbanization rate of resident population	0.514	0.155	0.196	0.896
ISU	1 × Primary industrial added value/GDP + 2 × Secondary industrial added value/GDP+ tertiary industrial added value/GDP	2.336	0.134	2.069	2.834
ACS	Ratio of sown area of grain crops to total sown area of crops	0.658	0.132	0.354	0.971
DISA	Ratio of disaster area to sown area	0.231	0.162	0.000	0.936
MCI	Ratio of total sown area of crops to total area of cultivated land	1.424	0.507	0.488	2.848
FSFA	Ratio of agricultural financial expenditure to total financial expenditure	0.097	0.036	0.010	0.204
MECH	Ratio of total power of agricultural machinery to output of planting industry	4.074	2.126	1.083	11.781

**Table 4:** AEE in 30 Chinese provinces in major years.

Province	2000	2005	2010	2015	2020	Average
Beijing	1.1656	1.1626	1.1724	1.1548	1.0293	1.1442
Tianjin	0.7608	0.6433	0.6531	0.6994	1.0719	0.7599
Hebei	0.6812	0.5796	0.6479	0.6358	0.8451	0.6443
Shanxi	0.5009	0.4578	0.4866	0.4603	0.5811	0.4871
Inner Mongolia	0.7637	0.6224	0.639	0.6651	0.7752	0.6568
Liaoning	1.0190	1.0168	1.0173	1.0523	1.0737	1.0322
Jilin	1.0677	1.1062	1.0615	1.1165	1.1452	1.1060
Heilongjiang	1.0736	1.0263	1.1363	1.1431	1.1595	1.0953
Shanghai	1.1317	1.1071	1.2110	1.0842	1.0132	1.0978
Jiangsu	0.8037	0.7034	0.8012	1.0127	0.8645	0.8142
Zhejiang	1.0120	0.6416	0.6377	0.6438	1.1125	0.7507
Anhui	0.5537	0.4638	0.5429	0.5358	0.5903	0.5091
Fujian	0.6144	0.6275	0.6813	0.6909	1.0417	0.7126
Jiangxi	0.6955	0.5501	0.5657	0.6161	0.7026	0.5866
Shandong	1.0306	0.6868	0.7521	0.7496	1.0015	0.7709
Henan	1.0106	0.6834	0.7672	0.7358	0.8791	0.7602
Hubei	0.8098	0.5750	0.5296	0.5319	0.5649	0.5514
Hunan	0.6610	0.5466	0.5544	0.5134	0.5806	0.5335
Guangdong	1.0150	0.8263	0.7644	0.7889	1.0405	0.8375
Guangxi	1.0727	1.1226	1.1725	1.0875	1.1215	1.1223
Hainan	1.2293	1.1777	1.1873	1.1540	1.1508	1.1792
Chongqing	0.7502	0.6983	0.6876	0.6701	0.6791	0.6528
Sichuan	1.0172	0.7179	0.7089	0.6461	0.7155	0.6920
Guizhou	1.0450	0.6836	0.498	0.4903	0.6560	0.6098
Yunnan	1.0768	0.7222	0.7048	0.6426	0.7737	0.7610
Shaanxi	0.6135	0.6130	0.6194	0.5765	0.6286	0.5821
Gansu	0.6196	0.5499	0.5316	0.5300	0.6940	0.5703
Qinghai	0.4901	1.0195	0.4956	0.4217	1.0650	0.6287
Ningxia	0.5007	0.4238	0.4775	0.4600	0.5300	0.4628
Xinjiang	1.1486	1.0413	1.0926	1.0793	1.0639	1.0674
Eastern	0.9444	0.8156	0.8508	0.8614	1.0171	0.8711
Northeastern	1.0534	1.0498	1.0717	1.1040	1.1261	1.0778
Central	0.7053	0.5461	0.5744	0.5656	0.6498	0.5713
Western	0.8271	0.7468	0.6934	0.6608	0.7911	0.7096
Average	0.8645	0.7599	0.7599	0.7530	0.8717	0.7726

**Table 5:** Influencing factors of AEE.

Variable	Coefficient	Standard Error	Z-Statistic	Probability
URBAN	0.648	0.033	19.85	0.000 ***
ISU	0.154	0.035	4.36	0.000 ***
ACS	−0.072	0.030	−2.39	0.017 **
DISA	−0.026	0.012	−2.15	0.031 **
MCI	−0.025	0.006	−4.48	0.000 ***
FSFA	0.540	0.081	6.65	0.000 ***
MECH	0.004	0.002	2.39	0.017 **

Notes: **, *** denote statistical significance at 5% and 1%, respectively.

## Data Availability

Data will be available from the corresponding author on request.

## Appendix A. Agricultural Carbon Emission Sources and Coefficients

**Agricultural Material**	**Coefficient**
Fertilizer	0.8956 kg C∙kg^−1^
Pesticide	4.9341 kg C∙kg^−1^
Mulch	5.1800 kg C∙kg^−1^
Diesel	0.5927 kg C∙kg^−1^
Irrigation	266.4800 kg C∙hm^−2^

## Appendix B. CH4 Emission Coefficients of Different Rice Varieties in Chinese Provinces (Units: g∙m^−2^)

**Province**	**Early-Season Rice**	**Late-Season Rice**	**Mid-Season Rice**	**Province**	**Early-Season Rice**	**Late-Season Rice**	**Mid-Season Rice**
Beijing	0	0	13.23	Henan	0.00	0.00	17.85
Tianjin	0	0	11.34	Hubei	17.51	39	58.17
Hebei	0	0	15.33	Hunan	14.71	34.1	56.28
Shanxi	0	0	6.62	Guangdong	15.05	51.6	57.02
Inner Mongolia	0	0	8.93	Guangxi	12.41	49.1	47.78
Liaoning	0	0	9.24	Hainan	13.43	49.4	52.29
Jilin	0	0	5.57	Chongqing	6.55	18.5	25.73
Heilongjiang	0	0	8.31	Sichuan	6.55	18.5	25.73
Shanghai	12.41	27.5	53.87	Guizhou	5.1	21	22.05
Jiangsu	16.07	27.6	53.55	Yunnan	2.38	7.6	7.25
Zhejiang	14.37	34.5	57.96	Shaanxi	0	0	12.51
Anhui	16.75	27.6	51.24	Gansu	0	0	6.83
Fujian	7.74	52.6	43.47	Qinghai	0	0	0.00
Jiangxi	15.47	45.8	65.42	Ningxia	0	0	7.35
Shandong	0.00	0.00	21.00	Xinjiang	0	0	10.50

## Appendix C. N2O Emission Coefficients of Soil From All Varieties of Crops (units: kg∙hm^−2^)

**Crop**	**N2O Emission Coefficients**
Rice	0.24
Spring Wheat	0.40
Winter wheat	2.05
Soybeans	0.77
Maize	2.532
Vegetables	4.21

## Appendix D. Economic Coefficient and Carbon Sequestration Rate of Main Crops in China

**Crop**	**Economic Coefficient**	**Moisture Content/%**	**Sequestration Rate**	**Crop**	**Economic Coefficient**	**Moisture Content/%**	**Sequestration Rate**
Rice	0.489	12	0.414	Yams	0.667	70	0.423
Wheat	0.434	12	0.485	Sugar cane	0.750	50	0.450
Core	0.438	13	0.471	Beet	0.667	75	0.407
Beans	0.425	13	0.450	Vegetables	0.830	90	0.450
Rapeseed	0.271	10	0.450	Melons	0.700	90	0.450
Peanut	0.556	10	0.450	Tobacco	0.830	85	0.450
Sunflower	0.300	10	0.450	Other crops	0.400	12	0.450
Cotton	0.100	8	0.450				
